# Revealing urban residents’ ecosystem service preferences in China: Evidence from a nationwide survey

**DOI:** 10.1038/s41597-026-06689-3

**Published:** 2026-02-09

**Authors:** Shuyao Wu, Delong Li, Lumeng Liu, Zhonghao Zhang

**Affiliations:** 1 College of Geography and Remote Sensing, Hohai University, Nanjing, Jiangsu 211100 China; 2 Institute of Geographic Sciences and Natural Resources Research, Chinese Academy of Science, Beijing, 100101 China

**Keywords:** Ecosystem services, Psychology and behaviour

## Abstract

Characterizing ecosystem services demand (ESD) is key to understanding the diverse preferences for various benefits from nature. However, direct evidence of the variations in ESDs among different groups of people and places remains limited. Here, a national-scale dataset of ESDs derived from a non-probabilistic survey of 20,075 urban residents across 31 provinces in China is presented. The dataset captures preferences for nine typical urban ecosystem services using a point-allotment experiment, where participants allocated a total of 100 importance points to prioritize ESDs. Key findings reveal significant variations in ESDs, with air purification receiving the highest average importance point (22.17), followed by recreation (15.60) and local climate regulation (13.62). This pattern of variation in ESDs is evident in 28 of 31 provinces. The dataset also includes detailed socioeconomic and environmental metadata, enabling further analyses of regional disparities and their drivers among ESDs. This resource offers exploratory insights into tailoring urban design and ecosystem management strategies to diverse societal needs, thereby advancing sustainable land use planning and ESD research.

## Background & Summary

Ecosystem services demand (ESD) refers to the needs, desires, and requirements of humans for the benefits provided by nature, reflecting the diverse preferences of different groups of people for various benefits^1,2^. It is an important concept that can contribute to fields such as land use planning^3,4^, natural resource management^5,6^, and urban park design^7,8^ through the incorporation of human needs. Studies have begun to utilize remotely sensed data, expert knowledge, and census indicators as proxies to understand ESD and have found significant variations among different ESDs^3^. For example, Cao *et al*.^4^ used expert knowledge to examine the spatial heterogeneity in ESD in the North China Plain and found great variations in ESDs within different land cover; Zhai *et al*.^6^ proposed a five-level ESD classification system based on the Hierarchy of Needs theory and exemplified the distinct spatial heterogeneity of six ESDs (i.e., grain production, water yield, carbon sequestration, soil conservation, habitat quality, and leisure services) in the Yellow River Basin using remotely sensed and census data; De Knegt *et al*.^5^ assessed the demand dynamics for 17 ESs in the Netherlands from 2000 to 2020 and reported great variations in the changing trends among the services. Despite these efforts, the current ESD study results still face problems, including low quantifiability, limited coverage, and high subjectivity. It remains challenging to find reliable ESD data to determine whether people from different places value various services the same way^8^. Direct, quantifiable evidence (e.g., survey or interview data) on ESD at a large scale is greatly needed to further advance relevant research and support decision-making.

Urban parks provide various ecosystem services to people, which are highly demanded by the growing urban population worldwide^7,9^. As urbanization accelerates globally, the demand for ecosystem services in urban areas has increased significantly to address issues like urban heat islands, flooding, and biodiversity conservation^10–12^. While much research has focused on quantifying the supply of ecosystem services in urban parks, there is a growing recognition of the necessity to assess service demand to ensure that urban parks meet the diverse needs of urban residents^13–15^. Studying ESD among urban parks provides an excellent example for understanding ESD variations among people from different cities. Therefore, this study used a national-scale survey and a point-allotment experiment to understand the potential variations among ESDs from urban parks across China and address the aforementioned important knowledge gap. The resulting data could provide a reproducible reference for the differences in ESDs at a large scale, enabling regional variation analyses and demand-based policy-making.

## Methods

### Questionnaire design

Following a concise introduction outlining the study’s objectives and informed consent for participation and data sharing, the online survey questionnaire was systematically organized into three distinct sections (please see Supplementary Information for details, also available at 10.5281/zenodo.17309624)^16^. The first section aimed to collect respondents’ demographic characteristics, encompassing socioeconomic data such as gender, age, income level, education level, and city of residence (questions Q1 to Q5). The second section focused on personal preferences, including self-reported interest in various environmental issues, monthly frequency of park visits, the size of the most frequently visited urban park, and self-assessed overall satisfaction with the natural environment in urban parks (questions Q6 to Q9). These factors obtained from sections 1 and 2 have been found to be able to influence ESDs in urban parks both theoretically and empirically^17–19^. The third section was designed to capture information on ecosystem service experiences that visitors perceived as actually enjoyed (i.e., services that respondents really experienced), wishfully enjoyed (i.e., services that respondents wish to experience), and a service importance point-allotment experiment (questions Q10 to Q12). For analytical purposes, the categorical variable “gender” (Q1) was coded as 1 for males and 2 for females. Ordinal variables (Q2 to Q9) were assigned numerical codes: age, income, education level, park visit frequency, and natural environment satisfaction using a 1 to 5 scale; the most frequently visited park size employed a 1 to 4 scale; and environmental issue interest levels utilized a 1 to 10 scale.

**Fig. 1:**
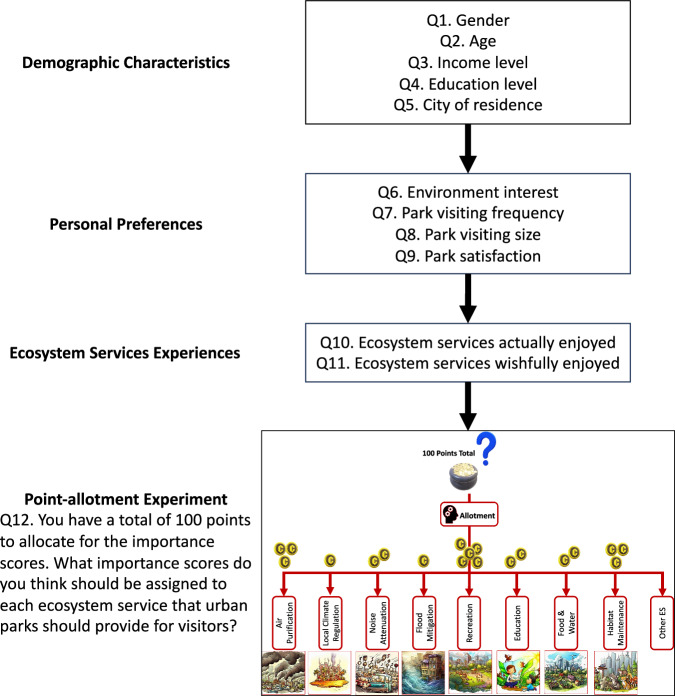
Flowchart of the survey design with an illustration of the point-allotment experiment designed to measure the variations among ecosystem services demands. Image of each service was drawn by DALL-E.

The importance point-allotment experiment (Q12) is the key to quantifying the potential variations among ESDs (Fig. 1). Respondents were provided with a total of 100 points to distribute across nine typical urban park ecosystem services: Air Purification, Local Climate Regulation, Noise Attenuation, Flood Mitigation, Recreation, Education, Food and Water Supply, Habitat Maintenance, and an “Others” category (defined by respondents). These services cover all provisioning, regulating, and cultural ES types and are among the most commonly studied urban ES worldwide^7,9^. Participants were instructed to allocate more points to services they deemed more important or with stronger perceived demand, with individual service allocations ranging from 0 to 100 points and the cumulative total not exceeding 100. For instance, equal importance would result in an average of approximately 11 points per service, whereas stronger demand for specific services could be reflected in disproportionately higher allocations at the expense of others. These examples were also provided to survey respondents, and a warning was given when the total points exceeded or fell short of 100 to ensure the reliability of responses. Unlike conventional Likert-scale approaches for assessing ecosystem services importance^20–22^, which measure absolute importance perceptions without accounting for trade-offs and may obscure priority differences, our method explicitly requires respondents to prioritize service importance by imposing a strict constraint on total point allocation, thereby highlighting potential differences in perceived value. Furthermore, compared with ranking methods, which capture only ordinal relationships, and discrete choice frameworks, which focus on binary or multiple-choice trade-offs among limited service combinations^23,24^, the point-allotment approach quantifies both the order and intensity of preferences, making it particularly suitable for analysing the relative importance of multiple interrelated ecosystem services.

### Data collection

Between July 2022 to January 2024, questionnaire data were collected through a systematic process, with ethics approval obtained from the Institutional Review Board of the College of Geography and Remote Sensing, Hohai University (20250624001). The online questionnaire underwent initial pilot testing via the authors’ personal social media networks (WeChat and Weibo; 328 responses in total) to refine its structure and clarity (e.g., the order of the questionnaire sections was adjusted, and explanatory notes were added to each question). Following the finalization of the questionnaire, data collection was facilitated by the commercial online survey platform Wenjuanxing (https://www.wjx.cn/), which was commissioned to recruit a diverse sample of urban residents across China. The Wenjuanxing sampling pool comprises over 2.6 million respondents, whose personal information has been verified, enabling authentic, representative, and diverse survey studies^25^. A minimum of 28,000 random responses were required from the commercial service of Wenjuanxing (each province was required to have a minimum of ten responses to ensure the national-scale coverage; survey respondents were restricted to the urban population; no specific requirement was made on other factors, such as respondents’ gender, age, occupation, income, or education). In this study, an ‘urban resident’ is operationally defined as an individual who self-reports their place of residence as a city at the municipal level. This definition covers built-up areas of cities at all administrative levels but does not distinguish between core urban areas and peri-urban contexts. It should also be noted that the dataset adopted a non-probabilistic sampling approach, and its representativeness is limited to the surveyed population rather than the broader national urban resident population. This partnership yielded a total of 29,248 initial responses, of which 28,920 were from the commercial service. Only data from individuals who provided consent to participate and to share their data were collected. To protect participants’ information, sensitive personal data, such as names and addresses, was not collected via the questionnaire. During data entry, encrypted coding is also employed, and an access permission system is implemented to ensure that only researchers with approval can access the original data. Data analysis is also strictly restricted to the scope of the research objectives.

Prior to further analysis, a rigorous screening process was implemented using five exclusion criteria to ensure data integrity:

1. Response times shorter than 100 seconds, indicating potential rushed or inattentive answering (the threshold value was determined by the minimum average answering time, ~100 to 120 seconds, recommended by the Wenjuanxing platform);
2. Response times exceeding 1,800 seconds, suggesting atypical or disengaged participation (the threshold value is approximately 8.85 standard deviations away from the mean answering time, which is 223.52 seconds);
3. Contradictory answers in Question 10, where respondents simultaneously selected “no ecosystem services are felt” and at least one specific service;
4. Contradictions between Answers 11 and 12, defined as not selecting “Other service” in Question 11 but assigning a value greater than 10 to “Other service” in Question 12;
5. Failure to correctly answer randomly embedded quality check questions.

**Fig. 2:**
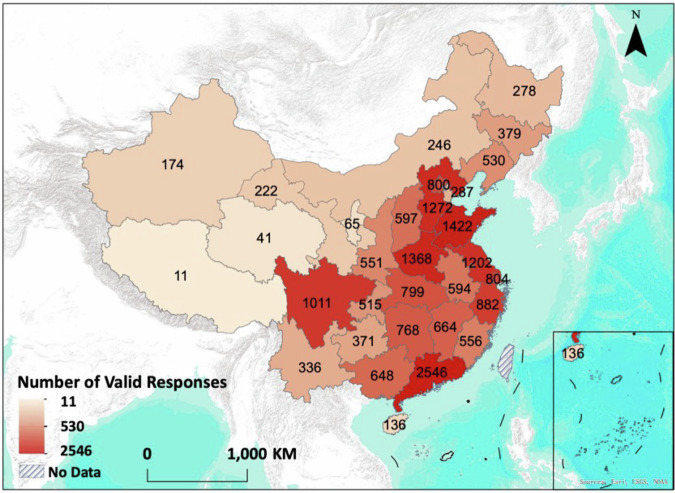
Number of valid responses across 31 provinces (or autonomous regions) across China.

After applying these criteria, 20,075 valid responses remained, representing 31 provinces or autonomous regions (Fig. 2) and 344 municipal-level cities in China^16^. The provinces of Guangdong, Shandong, Henan, Hebei, Jiangsu, and Sichuan all had more than 1,000 valid responses (Fig. 2). The average importance value and the corresponding standard deviation of each service in these provinces and cities were calculated to represent local ESD intensity and uncertainty, respectively.

## Data Record

The full survey results, including the socioeconomic, personal, and ESD data, are available at 10.5281/zenodo.17309624 and contained within the Excel file named ‘SurveyData.xlsx’^16^. This file is organized into four sheets, namely “SurveyData”, “SensitivityAnalysisData”, “Provincial level”, and “Municipal level”.

### SurveyData sheet

This sheet consists of 20,075 rows and 45 columns. The ‘No.’ column serves as a sequential identifier for each survey entry. ‘SubmitTime’ records the date and time when the survey was submitted, while ‘AnswerTime’ represents the time taken by respondents to complete the survey. Columns prefixed with ‘Q’ correspond to various survey questions and answers.

### SensitivityAnalysisData sheet

This sheet has 2,233 rows and 45 columns. All of its column names match those in the ‘SurveyData’ sheet, including ‘No.’, ‘AnswerTime’, and the ‘Q’-series of questions. However, the ‘AnswerTime’ of survey respondents in this sheet is either less than 100 seconds (N = 2,173) or greater than 1,800 seconds (N = 60). Therefore, the data in this sheet were only used in sensitivity analyses

### Provincial level sheet

This sheet contains 33 rows and 23 columns. The ‘Provinces’ column lists the names of provinces. ‘Count’ is a column that represents the count of the number of responses from that province. The columns named after various ecosystem services (i.e., Air Purification, Local Climate Regulation, Noise Attenuation, Flood Mitigation, Recreation, Education, Food and Water Supply, Habitat Maintenance, and Others) represent the averages and standard deviations related to these services at the provincial level.

### Municipal level Sheet

With 343 rows and 23 columns, this sheet is similar in structure to the ‘Province-level’ sheet but focuses on cities. The ‘Cities’ column lists the names of municipalities. ‘Count’ represents the count of the number of survey responses from that city. The columns named after various ecosystem services (i.e., Air Purification, Local Climate Regulation, Noise Attenuation, Flood Mitigation, Recreation, Education, Food and Water Supply, Habitat Maintenance, and Others) represent the averages and standard deviations related to these services at the municipal level.

### Data overview

**Fig. 3:**
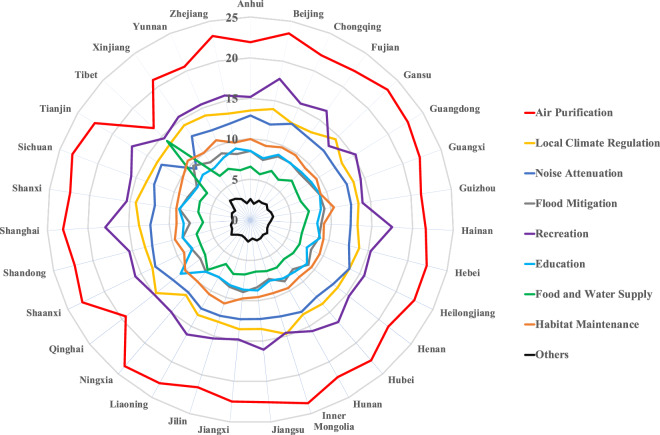
Average importance point of nine ecosystem services demands allotted by urban residents among 31 provinces (or autonomous regions) in China. Provinces (or autonomous regions) are ranked from the highest annual total GDP in 2024 (Guangdong) to the lowest (Tibet) in the clockwise direction.

The overall majority of the socio-economic and personal variables of all 20,075 respondents are women (54.76%), between 21 to 35 years old (70.09%), with bachelor degrees (66.15%), earn 5,001 to 10,000 CNY/month (39.17%), with an interest level of environmental issues 8 out of 10 (26.68%), most frequently visits medium-sized park (50.70%), and visits parks 6 to 15 times/month (66.62%) (Table 1). The importance point of each of the nine services was averaged across 31 provinces (or autonomous regions) (Fig. 3) and 344 municipal-level cities in China. At the province level, the service of Air Purification received the highest mean importance points (with an average of 22.17), followed by Recreation (with an average of 15.60), Local Climate Regulation (with an average of 13.62), Noise Attenuation (with an average of 12.39), Habitat Maintenance (with an average of 9.71), Education (with an average of 8.48), and Flood Mitigation (with an average of 8.46) (Fig. 3). The high importance points of Air Purification service could reflect a strong air pollution awareness and health concerns among the urban residents of China, and are consistent with the findings reported in more developed countries such as Singapore^21,26,27^. The service of Food and Water Supply received the lowest importance points (with an average of 6.88, except for Others) in 29 out of 30 provinces or autonomous regions, which indicates this service only has limited direct relevance to Chinese urban residents’ daily experiences in general (Fig. 3). However, the average importance points (14.18) of Food and Water Supply ranked the third highest in Tibet Autonomous Region, likely resulting from the abundant resources of wild edible plants and fungi used by the local Tibetan community^28,29^ (Fig. 3).

**Table 1:** Distribution of the seven socioeconomic and personal factors among 20,075 respondents.

Factors	Data Distribution					
Gender	Male				Female	
	45.24%				54.76%	
Age (years old)	<20	21–35	36–50	51–65	>66	
	9.32%	70.09%	18.29%	2.12%	0.18%	
Education Levels	Middle school or below	High school	College	Bachelor	Graduate school	
	1.88%	7.69%	15.41%	66.15%	8.88%	
Income Levels (CNY/month)	<3,000	3,001–5,000	5,001–10,000	10,001–15,000	>15,001	
	18.04%	21.45%	39.17%	14.68%	6.65%	
Environmental Issue Interests	0–2	3–4	5–6	7–8	9–10	
	3.66%	11.25%	28.43%	39.59%	17.06%	
Common Park Visit Size	Small	Medium	Large	Very large		
	50.70%	31.95%	12.17%	2.97%		
Park Visit Frequency (time/month)	<1	1–5	6–15	16–30	>30	
	14.24%	66.62%	15.86%	2.83%	0.45%	
Park Visit Satisfaction	Very dissatisfied	Dissatisfied	General	Satisfied	Very satisfied	Do not know
	0.71%	1.90%	26.69%	57.22%	13.14%	0.33%

## Technical Validation

**Fig. 4:**
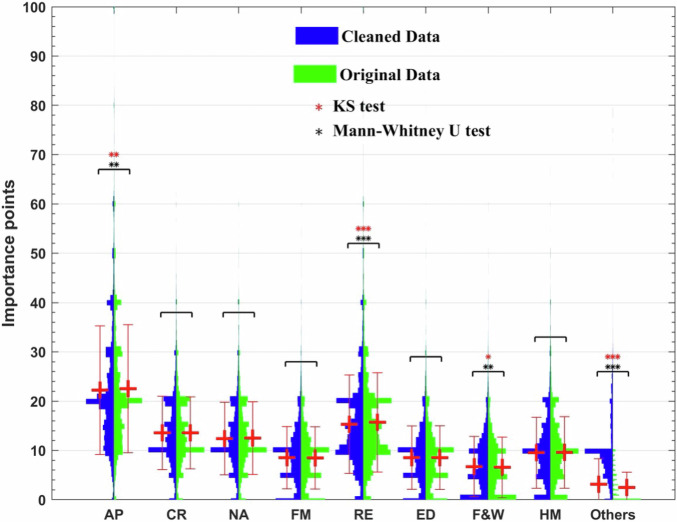
Differences in the importance points of nine ecosystem services demands from urban parks between the original (20,075) and extended (22,308) datasets. AP stands for air purification; CR stands for local climate regulation; NA stands for noise attenuation; FM stands for flood mitigation; RE stands for recreation; ED stands for education; F&W stands for food and water supply; HM stands for habitat maintenance. *P < 0.05, **P < 0.01, ***P < 0.001 based on Mann-Whitney U test and two-sample Kolmogorov-Smirnov test.

To assess the sensitivity of our survey findings, an additional analysis was conducted by reintegrating 2,233 excluded responses with extreme response times (either <100 seconds or >1,800 seconds) and examining their potential influence on variation among ESDs. The non-parametric Mann-Whitney U test and the two-sample Kolmogorov-Smirnov test were used to compare the mean and distribution differences in the importance points between the nine ecosystem services of the final, cleaned dataset (with 20,075 responses) and the original dataset (with 22,308 responses), respectively. The Kolmogorov-Smirnov test returns a test decision for the null hypothesis that the data in two vectors are from the same continuous distribution^30^. Both tests indicate that five out of nine ecosystem services (i.e., Local Climate Regulation, Noise Attenuation, Flood Mitigation, Education, and Habitat Maintenance) do not exhibit statistically significant differences in the average importance points and data distribution between the cleaned and original datasets, suggesting high robustness of the findings (Fig. 4).

## Usage Notes

To the best of our knowledge, this dataset contains the first national-scale (covering 31 provinces and 344 cities) survey results on ESDs, featuring a large sample size (20,075) and a quantitative importance point-allotment approach^16^. It is valuable for understanding variations in ESDs among the growing urban population and for providing exploratory support for analysing the preference characteristics of the surveyed population and generating hypotheses about people’s preferences for different services across China. When assessing the integrated importance of multiple ecosystem services, current approaches often assign uniform weights to various ecosystem services, failing to account for potential differences in preferences across service types^31–33^. Based on a large-scale questionnaire survey, this dataset can provide a possible reference when setting preference weights for different ecosystem services. These results will also provide insights for ecosystem services researchers, land-use planners, and urban park designers, supporting the development of hypotheses for management and planning strategies tailored to diverse ecosystem service demands. For instance, variations in ESDs can be incorporated into urban spatial prioritization and regional planning to optimize residents’ satisfaction. However, it should be acknowledged that the collected responses were skewed toward young-to-middle-aged, college-educated, and middle-income groups, and were obtained primarily from more developed regions. Cautions are thus necessary to ensure responsible reuse of the dataset, especially when underrepresented groups and regions are involved. It is recommended to combine this dataset with probabilistic survey data or census statistics for cross-validation in region-specific or national-scale research. In addition, another limitation is the inability to distinguish core urban from peri-urban residents within the self-reported ‘city of residence’, which could introduce uncertainty into fine-scale spatial comparison analyses and warrants more careful distinction in future studies.

## Supplementary information

Questionnaire

## Data Availability

The full survey results, including the socioeconomic, personal, and ecosystem service demand data, are available at 10.5281/zenodo.17309624.

## Code availability

The code for performing the Mann-Whitney U test, two-sample Kolmogorov-Smirnov test, and visualization is available at 10.5281/zenodo.17309624. The code was edited using MATLAB 9.4 (R2018a).
