# Supplementary material for: Revealing urban residents’ ecosystem service preferences in China: Evidence from a nationwide survey
Source: Sci Data. 2026 Feb 9;13:394. doi: 10.1038/s41597-026-06689-3 (PMC12996412; doi:10.1038/s41597-026-06689-3)
Supplement: Supplementary file 1 — Questionnaire [file 41597_2026_6689_MOESM1_ESM.docx]

**Supplementary Information**

**Translated version of the original questionnaire (and informed consent form) in English**

**Research Project: Survey on Ecological Service Needs of Urban Parks in China**

**Principal Researcher**
Name: Wu Shuyao
Affiliation: Shandong University
Contact Phone: 13917057134
Email: wushuyao@sdu.edu.cn

**Research Purpose**We hereby invite you to participate in a survey study. Before deciding to join this research, please ensure you understand the purpose of the study and what it will involve. Please read the following information carefully. If any information is unclear or you need more details, please consult the researchers. The purpose of this study is to understand your differences in demand for various natural ecological services provided by urban parks. The results will be used to put forward improvement suggestions for the planning and construction of urban parks.

**Research Process**This study will be conducted in the form of an online survey. During the survey, you will need to fill in some basic personal information. Then, you need to answer several questions to indicate your attitudes toward the natural ecological services provided by various urban parks. The entire survey takes approximately 5 minutes to complete.

**Research Risks**Being asked about your personal information may make you feel some discomfort, but there are no known risks in completing this survey. You may refuse to answer some or all of the questions. If you wish, you can terminate the questionnaire at any time.

**Survey Confidentiality**
Your responses to this questionnaire will be kept confidential. Please do not disclose any identifying information in the questionnaire.

**Contact Information**If you have questions about this study or encounter any adverse effects due to your participation, you can contact the researchers, whose information is provided above. If you have questions about your rights as a research participant, please contact Wu Shuyao at 13917057134.

**Voluntary Participation**
Your participation in this study is voluntary. The decision to participate is up to you. If you decide to participate, you will be required to sign an informed consent form. After signing the consent form, you can still withdraw at any time for any reason, and withdrawing from the study will not affect your relationship with the researchers.

**Informed Consent Form**
I have read and understood the provided information and have had the opportunity to ask questions. I voluntarily participate in the survey and can withdraw at any time for any reason.
If you agree and acknowledge the above content, please click the "Agree" button below to start answering the questions. If you do not agree or acknowledge the above content, please click the "Disagree" button below to interrupt this questionnaire survey project.

**Study on the Demands for Ecosystem Services in Urban Parks in China**

Hello! We are the joint research team of Chinese Academy of Sciences-Shandong University on the ecosystem service needs of urban parks. We would like to understand the differences in your demands for different natural ecosystem services in various types of urban parks, and the results will be used to make suggestions for improvement in the planning and construction of urban parks. This questionnaire contains 12 questions. Thank you very much for your support!

Q1. What is your gender: [Single-Choice]

| ○Men | ○Women |  |  |  |  |  |  |
| --- | --- | --- | --- | --- | --- | --- | --- |

Q2. What is your age group: [Single-choice]

| ○20 years old and below |
| --- |
| ○21-35 years old |
| ○36-50 years old |
| ○51-65 years old |
| ○66 years old and above |

Q3. What is your **usual** city of residence: [Fill in the blank]

_________________________________

Q4. What is your education level? [Single-choice]

| ○Junior high school and below |
| --- |
| ○High school |
| ○College |
| ○Bachelor's degree |
| ○Graduate students and above |

Q5. What is your net monthly income? [Single-choice]

Note: "net income" refers to after-tax, or actual disposable income earned

| ○3000 RMB/month or less |
| --- |
| ○3001-5000 RMB/month |
| ○5001-10000 RMB/month |
| ○10001-15000 RMB/month |
| ○15001 RMB/month or more |

Q6. What is your usual level of concern about **environemental issues**? [Enter a number from 0 to 10]

Note: A score of 10 means very concerned (e.g. try to understand all the ecological issues you come into contact with), 0 means never concerned

|  |  |
| --- | --- |
| Level of Concern | ________________________ |

Q7. Please select the two types of urban parks you visit most often in your spare time (**1 being the most often type**, **2 being the second most often type**) [ranking questions, please enter the numbers in order in the brackets]

Note: If you have not visited any of the parks listed below, **please select only "Have not visited any of the above park types"**.

| [ ] Small parks (the max time required to visit is about half an hour): such as amusement parks, community parks, etc. |
| --- |
| [ ] Medium-sized parks (the max time required to visit is half to 2 hours): such as comprehensive parks, riverfront parks, large community parks, etc. |
| [ ] Large parks (the max time required to visit is up to 2 to 5 hours): such as forest parks, wetland parks, botanical gardens, etc. |
| [ ] Very large parks (the max time required to visit is greater than 5 hours): such as large country parks, wildlife parks, scenic spots, etc. |
| [ ] Have not visited to any of the above park types, or have only been to parks with less natural environment, such as green areas along the street, green areas within the district, commercial green areas, amusement parks, etc. |

Q8. What is the average number of times you visit urban parks (**including all the types of parks selected in the previous question**) in spare time per month? [Single-choice]

| ○Less than 1 time/month |
| --- |
| ○1-5 times/month |
| ○6-15 times/month |
| ○16-30 times/month |
| ○30 times/month or more |

Q9. What is your overall satisfaction level with the **natural environment of** the various parks in your city? [Single-choice]

Note: "Natural environment" refers to animals, plants, water bodies, etc. in the park, excluding man-made facilities (such as roads and amusement facilities) in the park.

| ○Very dissatisfied |
| --- |
| ○Dissatisfied |
| ○General |
| ○Satisfied |
| ○Very satisfied |
| ○Never been there, don't know |

Q10. Please select the ecosystem services that you **actually felt** in various urban parks? [Multiple-choice]

| □ Air Purification: The leaves of plants in the park absorb pollutants and dust in the atmosphere, reducing the content of toxic substances and suspended particles in the air |
| --- |
| □ Local Climate Regulation: The vegetation in the park regulates the temperature and humidity in the park during the hot summer months through shading and evapotranspiration of the trees. |
| □ Noise Attenuation: The vegetation in the park reduces noise from surrounding traffic, buildings and domestic sources |
| □ Flood Mitigation: Vegetation in the park can reduce the runoff from rainwater and reduce the workload of the urban drainage system |
| □ Recreation: The park can be used as a place for people to enjoy the natural scenery and relax. |
| □ Education: The park can provide a place for people to learn about and study nature |
| □ Food and Water Supply: Food such as wild fruits and vegetables grown in the park can be picked and eaten, and clean water can be used |
| □ Habitat Maintenance: The park provides habitat for urban flora and fauna, and contributes to the pollination of species within the city |
| □ Other: Please add any ecosystem services you think should be provided after checking |
| □ No ecosystem services are felt |

Q11. Please select the ecosystem services that you think various types of urban parks **should** provide for visitors? [Multiple-choice]

Note: Unlike the previous question, this question wants to know **whether** you **approve of the services you have experienced** and **whether there are services you would like to receive but have not actually received.**

| □ Air purification: The leaves of plants in the park absorb pollutants and dust in the atmosphere, reducing the content of toxic substances and suspended particles in the air |
| --- |
| □ Local climate regulation: The vegetation in the park regulates the temperature and humidity in the park during the hot summer months through shading and evapotranspiration of the trees. |
| □ Noise attenuation: The vegetation in the park reduces noise from surrounding traffic, buildings and domestic sources |
| □ Flood mitigation: Vegetation in the park can reduce the runoff from rainwater and reduce the workload of the urban drainage system |
| □ Recreation: The park can be used as a place for people to enjoy the natural scenery and relax. |
| □ Education: The park can provide a place for people to learn about and study nature |
| □ Food and water supply: Food such as wild fruits and vegetables grown in the park can be picked and eaten, and clean water can be used |
| □ Habitat maintenance: The park provides habitat for urban flora and fauna, and contributes to the pollination of species within the city |
| □ Other: Please add any ecosystem services you think should be provided after checking |

Q12. Weighting Question (you can slide or fill in the score manually): You have a total of 100 points to allocate for the importance scores. Could you please tell me what importance scores you think should be assigned to each ecosystem service that urban parks should provide for visitors? [Weighting question]

Note: The **total score for each ecosystem service must be equal to 100** (you can see the sum of the scored values after the question). A higher score means that the service is considered more important, a score of 0 means that there is no service, and a score of the same means that the service is considered equally important.
Example: If the first 8 ecosystem services are considered to be of equal importance, you can make each service score 12 or 13 by sliding or filling them in (**if the previous question is not filled in with "other" services, the score for "other" should be 0**).

Air Purification ________________________

Local climate regulation ________________________

Noise attenuation ________________________

Flood mitigation ________________________

Recreation ________________________

Education ________________________

Food and water supply ________________________

Habitat maintenance ________________________

Other (i.e., any ecosystem services you have filled in the previous question) ________________________

Hint: Please fill in the numbers; the sum of all items must be equal to 100

Random Quality Check Question:

1. “Where is the capital of China”, which word appeared in this sentence：[Single-Choice]

| ○Beijing |
| --- |
| ○Shanghai |
| ○Nanjing |
| ○Guangzhou |
| ○Capital |

2. In the four cities of “Bei-Shang-Guang-Shen”, which city does “Guang” stand for: [Single-Choice]

| ○Guanggao |
| --- |
| ○Guangyuan |
| ○Guangzhou |
| ○Guangan |

**Original version of questionnaire (and informed consent form) in Chinese**

**研究项目：中国城市公园生态服务需求调查研究**

**主要研究者**

姓名：吴舒尧

单位：山东大学

联系电话：13917057134

邮箱：wushuyao@sdu.edu.cn

**研究目的**

我们在此邀请您参与一项调查研究。在您决定参加这项研究之前，请务必确认进行此项研究的目的以及它将涉及的事项。请仔细阅读以下信息，如有不清楚的信息或是需要更多的信息请询问研究人员。本研究的目的是希望了解您对各类城市公园不同的自然生态服务需求差异，结果将用于对城市公园的规划与建设提出改进建议

**研究过程**

这项研究将以在线调查的形式进行。在调查中，您将需要填写一些基本的个人信息。然后，您需要回答几个问题来表明您对于各类城市公园提供的自然生态服务的态度。整个调查大约需要5分钟才能完成。

**研究风险**

被问及您的个人信息可能会使您感到一些不适，但完成此调查没有任何已知风险。您可以拒绝回答一些或所有的问题。如果您愿意，您可以随时终止您的问卷填写。

**调查保密性**

您对此问卷的回答将作保密处理。请不要在问卷中透露您的任何身份信息。

**联系方式**

如果您对本研究有疑问，或者您因参与本研究而遇到不利影响，都可以联系研究人员，研究人员信息在上文已经提供。如果您对作为研究参与者的权利有疑问，请联系吴舒尧13917057134。

**自愿参与**

您自愿参加这项研究。您是否参加这项研究将由您决定。如果您决定参加此研究，将被要求签署知情同意书。在签署同意书后，您仍然可以随时无理由退出，退出这项研究不会影响你与研究人员的关系。

**知情同意书**

我阅读并知晓所提供的信息，并有机会提出疑问。我自愿参与调查，且可以随时无理由退出。

如您同意并认可以上内容，请点击下面「同意」按钮，开始回答题目内容。如您不同意或不认可以上内容，请点击下面「不同意」按钮，中断本次问卷调研项目。

**中国城市公园生态服务需求调查研究**

您好！我们是中国科学院-山东大学城市公园生态服务需求联合调研团队。我们希望了解您对各类城市公园不同的自然生态服务需求差异，结果将用于对城市公园的规划与建设提出改进建议。本问卷共包含12题，非常感谢您的支持！

Q1. 请问您的性别是：[单选题]

| ○男 | ○女 |  |  |  |  |  |  |
| --- | --- | --- | --- | --- | --- | --- | --- |

Q2. 请问您的年龄段是：[单选题]

| ○20岁及以下 |
| --- |
| ○21-35岁 |
| ○36-50岁 |
| ○51-65岁 |
| ○66岁及以上 |

Q3. 请问您的**常住**城市是: [填空题]

_________________________________

Q4. 请问您的学历是？[单选题]

| ○初中及以下 |
| --- |
| ○高中或中专 |
| ○大专 |
| ○本科 |
| ○研究生及以上 |

Q5. 请问您每月的**到手**收入约是？[单选题]

注：“到手收入”指税后，或实际获得的可支配收入

| ○3000元/月以下 |
| --- |
| ○3001-5000元/月 |
| ○5001-10000元/月 |
| ○10001-15000元/月 |
| ○15001元/月以上 |

Q6. 请问您平时对**生态环境**问题的关注程度是？[矩阵文本题] [输入0到10的数字]

注：10分代表非常关注（例如接触到的所有生态环境问题均会尝试了解），0分代表从不关注

|  |  |
| --- | --- |
| 关注程度 | ________________________ |

Q7. 请选择您在空闲时最经常前往的两种城市公园类型（**1为最常去的类型**，**2为第二常去的类型**） [排序题，请在中括号内依次填入数字] *

注：如未去过下述各类公园，**请只选“未去过上述任何公园类型”一项**

| [ ]小型公园（最长游玩时间需约半小时）：如游园、社区公园等 |
| --- |
| [ ]中型公园（最长游玩时间需半-2小时）：如综合性公园、滨河公园、大型社区公园等 |
| [ ]大型公园（最长游玩时间需2-5小时）：如森林公园、湿地公园、植物园等 |
| [ ]超大型公园（最长游玩时间需大于5小时）：如大型郊野公园、野生动物园、风景名胜区等 |
| [ ]未去过上述任何公园类型，或仅去过如沿街绿化带、小区内绿地、商业绿地、游乐园等自然环境较少的公园 |

Q8. 请问您每月平均在空闲时前往各类城市公园（**含上题所选的所有公园类型**）的次数约为？ [单选题]

| ○小于1次/月 |
| --- |
| ○1-5次/月 |
| ○6-15次/月 |
| ○16-30次/月 |
| ○30次/月以上 |

Q9. 请问您对您所在城市的各类公园的**自然环境**总体满意度约为？ [单选题]

注：“自然环境”指公园中动物、植物、水体等，不含公园中的人造设施（如道路、游乐设施等）

| ○非常不满意 |
| --- |
| ○不满意 |
| ○一般 |
| ○满意 |
| ○非常满意 |
| ○从未去过，不了解 |

Q10. 请选择您在各类城市公园中**实际感受**到的生态服务有？ [多选题]

| □空气净化: 公园中的植物叶片能吸收大气中的污染物与粉尘, 降低空气中有毒物质和悬浮颗粒物含量 |
| --- |
| □温湿度调节: 公园中的植被在炎热的夏季通过树木的荫蔽和蒸腾作用调节公园内的温度和湿度 |
| □噪音消减: 公园中的植被能够降低来自周边交通、建筑与生活等来源的噪音 |
| □洪涝消减: 公园中的植被可以减轻雨水对土地的冲刷, 并减轻城市排水系统的工作强度 |
| □休闲赏景: 公园可以作为人们观赏自然风景、休闲放松的场所 |
| □知识教育: 公园可以为人们提供一个了解、学习大自然的场所 |
| □食物或水供给: 公园中生长的野菜与野果等食物可被采摘食用、清洁水可被使用 |
| □动植物保护: 公园为城市中的动植物提供栖息地和食物，并有助于城市内物种的授粉 |
| □其他: 勾选后请补充任意您实际感受到的生态服务 _________________ |
| □基本没有感受到任何生态服务 |

Q11. 请选择您认为各类城市公园**应该**为访客提供的生态服务有？ [多选题]

注：与上题不同，此题希望了解您**是否认可已感受到的服务**，以及**是否还有您希望获得但实际未获得的服务**

| □空气净化: 公园中的植物叶片能吸收大气中的污染物与粉尘, 降低空气中有毒物质和悬浮颗粒物含量 |
| --- |
| □温湿度调节: 公园中的植被在炎热的夏季通过树木的荫蔽和蒸腾作用调节公园内的温度和湿度 |
| □噪音消减: 公园中的植被能够降低来自周边交通、建筑与生活等来源的噪音 |
| □洪涝消减: 公园中的植被可以减轻雨水对土地的冲刷, 并减轻城市排水系统的工作强度 |
| □休闲赏景: 公园可以作为人们观赏自然风景、休闲放松的场所 |
| □知识教育: 公园可以为人们提供一个了解、学习大自然的场所 |
| □食物或水供给: 公园中生长的野菜与野果等食物可被采摘食用、清洁水可被使用 |
| □动植物保护: 公园为城市中的动植物提供栖息地和食物，并有助于城市内物种的授粉 |
| □其他: 勾选后请补充任意您认为应该提供的生态服务 _________________ |

Q12. 比重分配题(可滑动或手动填写得分)：您总共有100分可用于分配的重要性得分，请问您认为城市公园应该为访客提供的各项生态服务重要性得分应为？ [比重题]

注：**各项生态服务得分总和必须等于100分**（您可在题后看到已打分值总和）。给予分数越高代表认为此项服务越重要，给予0分代表认为没有此项服务，给予分数相同代表认为服务重要性相同。
例：如认为前8项生态服务重要性一致则可通过滑动或填写使每项服务得分为12或13分（**如上一题未填写“其他”服务，则“其他”项得分应为0**）。

空气净化________________________

温湿度调节________________________

噪音消减________________________

洪涝消减________________________

休闲赏景________________________

知识教育________________________

食物或水供给________________________

动植物保护________________________

其他（即您在上一题如有填写的任意生态服务）________________________

提示：请填入数字，所有项总和必须等于100

随机质量控制题：

1. “中国的首都是哪里？”下面那个词语出现在前面一句话中：[单选题]

| ○北京 |
| --- |
| ○上海 |
| ○南京 |
| ○广州 |
| ○首都 |

2. 北上广深的“广”代表哪个城市？：[单选题]

| ○广告 |
| --- |
| ○广元 |
| ○广州 |
| ○广安 |
